# Editorial: Mechanisms involved in the development of obesity with PCOS

**DOI:** 10.3389/fendo.2024.1444299

**Published:** 2024-07-01

**Authors:** Anju E. Joham, Negar Naderpoor, Ozlem Celik

**Affiliations:** ^1^ Monash Centre for Health Research and Implementation, Faculty of Medicine, Nursing and Health Sciences, Monash University, Melbourne, VIC, Australia; ^2^ Department of Endocrinology and Diabetes, Monash Health, Clayton, VIC, Australia; ^3^ Division of Endocrinology and Metabolism, Department of Internal Medicine, School of Medicine, Acibadem University, Istanbul, Türkiye

**Keywords:** obesity, weight, polycystic ovary syndrome, PCOS, insulin resistance

While polycystic ovary syndrome (PCOS) stands as the most prevalent endocrine disorder among women of reproductive age, its underlying pathophysiology remains incompletely understood. Various cytokines and adipokines have been proposed to contribute to the development of PCOS. In their recent article, Wang et al., introduced a novel approach encompassing mechanistic, cross-sectional, and interventional studies involving both animals and human subjects to investigate the involvement of glycoprotein β5 (GPHB5) in PCOS and insulin resistance. Their investigation demonstrated heightened tissue expression of GPHB5 mRNA in mice fed a high fat diet (HFD) compared to those on a normal chow diet (NCD), as well as in mouse models of obesity and diabetes in contrast to wild-type counterparts. In addition, they show an increased hepatic expression of GPHB5 mRNA in PCOS rat models post subcutaneous administration of DHEA. Correspondingly, a cohort of 483 women reveals elevated serum levels of GPHB5 in women with PCOS and those with insulin resistance (as determined by euglycemic hyperinsulinemic clamp) relative to controls. Furthermore, GPHB5 concentration is found to be positively correlated with various metabolic parameters. Notably, their interventional studies demonstrate a significant reduction in GPHB5 concentration concurrent with an increase in adiponectin level and insulin sensitivity after treatment with metformin, liraglutide and rosiglitazone in women with PCOS. These observations suggest, for the first time in human subjects, a potential involvement of GPHB5 in insulin resistance and PCOS. Nevertheless, future studies are warranted to further establish these findings and to determine if GPHB5 assumes a causative role in the pathophysiology of PCOS or if it may serve as a biomarker or therapeutic target.

Both PCOS and obesity are associated with insulin resistance and chronic low-grade inflammation. The complement system plays an important role in inflammation. In an innovative exploration, Ramanjaneya et al., compare the effects of moderate aerobic exercise over an 8-week period on insulin sensitivity and the complement system in 11 women with PCOS against 10 age and weight-matched controls. Despite observing an enhancement in insulin sensitivity and cardiometabolic fitness following exercise in both women with PCOS and controls, with no significant change in weight, the authors noted a more pronounced amelioration in several complement-related proteins among the control group compared with women with PCOS. They propose a dysregulation in the complement-system influencing the response to exercise in women with PCOS. Acknowledging the study’s limited sample size, the authors advocate for future studies with more participants allowing for control of co-variates, to validate these findings and elucidate their implications for PCOS management.

Yu et al. present the characteristics and possible mechanisms of metabolic disorder in overweight women with PCOS. The authors conducted a cross-sectional study on 947 women with PCOS, who were classified according to body mass index (BMI) as either overweight (BMI ≥ 24 kg/m2) or non-overweight (BMI ≤ 23.9 kg/m2). Increased indices of insulin resistance and a higher prevalence of acanthosis nigricans were both seen in the overweight group. There were no significant differences between the two groups (overweight and non-overweight) in terms of clinical and biochemical hyperandrogenism. A subcohort of eight adult patients with PCOS were randomly selected from overweight and non-overweight groups, and another 60 patients with PCOS (30 in each group) were randomly selected and had further testing. This revealed significantly different inflammatory cytokines between the overweight and non-overweight PCOS groups using ELISA, suggesting that overweight women with PCOS may have chronic low-grade inflammation.

Recent studies have shown that the prevalence of non-alcoholic fatty liver disease (NAFLD) in PCOS patients with obesity is significantly higher than that in the normal population. The Controlled Attenuation Parameter (CAP), which is based on the principle of instantaneous elastography, quantifies liver steatosis by measuring the attenuation of the ultrasound beam in direct correlation with liver fat content and non-invasive method. Wang et al. investigated the changes and influencing factors of liver CAP in obese patients with PCOS using FibroTouch and to determine the prevalence and risk factors of NAFLD in PCOS patients with obesity. They found that liver CAP was associated with disorders of lipid metabolism, insulin resistance, and hyperandrogenemia, with elevated testosterone levels being an independent risk factor for NAFLD in PCOS patients with obesity.

Medical therapy to improve metabolic profile in women with PCOS is an avenue of significant research. Whilst metformin has been well studied for this purpose, the study by Wen et al. examined the effect of combining beinaglutide with metformin compared with metformin alone over a treatment period of 12 weeks for improving metabolic profile. Beinaglutide is a short-acting recombinant human GLP-1 (rhGLP-1) and is an approved treatment for T2DM as per the Chinese Guideline for the Prevention and Treatment of T2DM. The study found that the combination therapy was favorable in terms of anthropometric measurements (body mass index, waist circumference and waist hip ratio). Participants in the combination arm lost greater amount of weight 4.54 ± 3.16 kg compared with a 2.47 ± 3.59 kg loss in the metformin alone arm. The most frequent adverse effects reported were gastrointestinal in the metformin alone arm and subcutaneous induration and local pruritis at the injection site in the combination arm.

Several studies have investigated the use of GLP-1 receptor agonists in improving favorable metabolic changes and significant weight loss in women with overweight or obesity and PCOS. But weight regain after weight loss is a dramatic problem due to the compensatory metabolic adjustments. Wilding JPH et al. showed that one year after withdrawal of 68 week intervention with semaglutide, participants regained 2/3 of their prior weight loss with residual benefits in some changes in cardiometabolic variables. Beyond glycemic control and weight reduction, GLP-1 receptor agonists have shown potential benefits on hormonal and reproductive parameters in PCOS. In this topic, Jensterle et al. investigated changes in body weight, cardiometabolic and endocrine parameters in obese women with PCOS who continued treatment with metformin 2 years after discontinuation of short-term intervention with semaglutide. They found that two years after semaglutide withdrawal, women with PCOS who continued with metformin regained about one-third of the semaglutide-induced weight loss. At the end of the follow up, 84% of women had a lower body weight than at baseline.

Further studies are warranted to study the potential role of GPHB5 in insulin resistance and PCOS and to further examine potential dysregulation in the complement-system influencing the response to exercise in women with PCOS. There is evidence of increased inflammatory cytokines in overweight women with PCOS. Liver elastography was associated with disorders of lipid metabolism, insulin resistance, and hyperandrogenemia, with elevated testosterone levels being an independent risk factor for NAFLD in PCOS patients with obesity. There are some promising results of GLP1 analogues in patients to improve metabolic profiles in women with PCOS, which warrant further long-term studies.

## Author contributions

AJ: Writing – original draft, Writing – review & editing. NN: Writing – original draft, Writing – review & editing. OC: Writing – original draft, Writing – review & editing.

